# Identification of the risk for liver fibrosis on CHB patients using an artificial neural network based on routine and serum markers

**DOI:** 10.1186/1471-2334-10-251

**Published:** 2010-08-24

**Authors:** Danan Wang, Qinghui Wang, Fengping Shan, Beixing Liu, Changlong Lu

**Affiliations:** 1 Institute of Immunology, China Medical University, Shenyang, Liaoning, China

## Abstract

**Background:**

Liver fibrosis progression is commonly found in patients with CHB. Liver biopsy is a gold standard for identifying the extent of liver fibrosis, but has many draw-backs. It is essential to construct a noninvasive model to predict the levels of risk for liver fibrosis. It would provide very useful information to help reduce the number of liver biopsies of CHB patients.

**Methods:**

339 chronic hepatitis B patients with HBsAg-positive were investigated retrospectively, and divided at random into 2 subsets with twice as many patients in the training set as in the validation set; 116 additional patients were consequently enrolled in the study as the testing set. A three-layer artificial neural network was developed using a Bayesian learning algorithm. Sensitivity and ROC analysis were performed to explain the importance of input variables and the performance of the neural network.

**Results:**

There were 329 patients without significant fibrosis and 126 with significant fibrosis in the study. All markers except gender, HB, ALP and TP were found to be statistically significant factors associated with significant fibrosis. The sensitivity analysis showed that the most important factors in the predictive model were age, AST, platelet, and GGT, and the influence on the output variable among coal miners were 22.3-24.6%. The AUROC in 3 sets was 0.883, 0.884, and 0.920. In the testing set, for a decision threshold of 0.33, sensitivity and negative predictive values were 100% and all CHB patients with significant fibrosis would be identified.

**Conclusions:**

The artificial neural network model based on routine and serum markers would predict the risk for liver fibrosis with a high accuracy. 47.4% of CHB patients at a decision threshold of 0.33 would be free of liver biopsy and wouldn't be missed.

## Background

Approximately 350 million people worldwide are chronically infected with hepatitis B virus (HBV), especially in many developing countries in Asia[1-3]. Liver fibrosis progression is commonly found in patients with chronic hepatitis B (CHB). Up to 40% of CHB patients will develop complications of liver cirrhosis and hepatocellular carcinoma (HCC)[4]. Patients with significant fibrosis or cirrhosis should be considered for antiviral therapy, which can potentially reverse cirrhosis and reduce complications[5-7].

Currently, liver biopsy, a widely used gold standard for the examination of the extent of liver fibrosis and HCC in patients with CHB, has many draw-backs, such as pneumothorax, pain, hemorrhage, or puncture of other viscera[8-10]. In addition, sampling error leads to only 65% accuracy in liver biopsy specimens[11]. Therefore, there is an increasing demand for noninvasive predictive models of liver fibrosis. Those methods could predict the risk probability of liver fibrosis and identify CHB patients at high risk for liver fibrosis. If we only perform liver biopsy on CHB patients at high risk for liver fibrosis, the number of liver biopsies would be reduced. In the past few years, noninvasive biochemical markers of liver fibrosis which could respond of the chronically injured liver had made considerable progression[12-14]. Multiple algorithms based on a combination of fairly routine parameters had been repeatedly suggested[15-17]. However, most of them required non-routine laboratory assays or used complex and patented models so that their regular use was limited. In fact, the true usefulness of any indices or models for application to clinical settings should be the easiness of the procedure and analytical simplicity, so that the results can be compared between laboratories for a long period[18].

The complete blood count and liver function are routine tests on CHB patients with hepatitis B surface antigen (HBsAg-positive) in the clinical treatment. It is easy to measure those markers with low cost in some laboratories, even in the Community Hospitals. Moreover, the artificial neural network (ANN) is potentially more successful than a traditional statistical model in predicting clinical outcome [19,20]. It can build nonlinear statistical models through learning examples and has been widely applied to predict, diagnose, and classify disease in many fields [21,22]. In our study, we designed a neural network which used a Bayesian learning algorithm by introducing probabilistic treatment of the Bayesian inference technique. It can overcome some difficult problems, such as local trapping, over-fitting, and overtime in training. Also, it is proposed to have significant advantages over the conventional neural network approach [23].

The present study was performed on HBsAg-positive patients. An artificial neural network model based on routine and serum markers was constructed to predict the risk for liver fibrosis. The objective of this study was to determine the feasibility of these indices of routine and serum markers to predict the risk for liver fibrosis. Furthermore, we aimed to similarly validate the probability value in order to identify the risk for liver fibrosis on CHB patients and classify high risk groups.

## Methods

### Patients

In April 2008, all HBsAg-positive in-patients who had liver biopsy between January 2006 and March 2008 were retrospectively investigated at the Hospital of Infectious Diseases. Only patients without the following conditions were included for the study: presence of other causes of liver disease such as chronic hepatitis C (CHC), or hepatitis E (HE), etc., acute hepatitis, hepatocellular carcinoma, prior liver transplantation, insufficient liver tissue for staging of fibrosis, and incomplete data on complete blood count or some serum markers of fibrosis. Additionally, patients without routine and serum markers prior to drug treatment were also excluded. Within a week of liver biopsy, those markers were recorded. If more than one set of laboratory results were available, the set of results closest to the time of biopsy were used. Later, additional in-patients who met the aforementioned criteria were consequently enrolled in the study from April 2008 to July 2009.

The enrolled patients were informed all procedures which conformed to the Helsinki Declaration. The study procedures were also approved by the Ethics Committee of the Infectious Diseases Hospital of Shenyang. The verbal informed consent for all patients was provided.

### Laboratory tests

Clinical chemistry tests were performed using 7150 Analyzer (Hitachi, Japan), and the complete blood count were measured on Hematology Analyzer (Beckman Coulter 5diff, U.S.A.).

### Liver biopsy histology

Liver biopsy was performed by automatic fare cut biopsy needle. Length and width of each sample were at least 10 mm and 1 mm, respectively. Liver sample of patients who were enrolled since April 2008 would contain 2 or more portal spaces. A single pathologist who had no clinical information of patients evaluated all biopsy results. The level of fibrosis was measured according to the METAVIR system, which had previously been applied in other reports on CHB[24,25]. Fibrosis was staged from F0 to F4: F0, no fibrosis; F1, portal fibrosis without septa; F2, few septa; F3, numerous septa without cirrhosis; and F4, cirrhosis. Generally, significant fibrosis was defined by F2, F3, or F4 stages; whereas F0 or F1 was considered as insignificant fibrosis.

### Statistical analysis

All statistical analysis was performed using MATLAB Neural Network Toolbox (2006) and SPSS Version 11.5 (SPSS, Inc, Chicago, IL). Data are reported as median (minimum-maximum) unless otherwise stated. The relationship between variables and the presence of significant fibrosis was assessed. The Mann-Whitney U Test, or Kruskal-Wallis Test was used for continuous variables when appropriate. The Chi-square test was used for categorical variables, and Fisher's exact test when appropriate. Those variables found to be strongly correlated to the presence of significant fibrosis were used to build the ANN.

### Development of the neural network

Patients between January 2006 and March 2008 were randomly divided into 2 subsets: training set and validation set, with twice as many patients in training set as in validation set. Patients since April 2008 formed the testing set. The three-layer neural network model was built and trained using a Bayesian learning algorithm, which had 1 output neuron (0, insignificant fibrosis; 1, significant fibrosis). To determine the optimal number of neurons in the intermediate layer, we randomly split all data into 5 subsets of equal size. For any given number of neurons, we trained the network on all but one subset, and tested it on the remaining one. We started from 6 neurons, and gradually increased the number of neurons. When there were more than 13 neurons, the performance of the trained neural network on output sample in the testing set began to deteriorate. Hence, the intermediate layer had 13 neurons in the current neural network model. The number of training epochs was set to 300, the learning rate was 0.05, and the training goal was set at 0.001.

As the neural network can be overtrained to recognize specific cases in the training set and result in good performance in the training set but not in the testing set, the validation set was used to decide when to stop training in order to minimize the potential bias.

### Sensitivity analysis

Neural network models have been long criticized for being black box solutions primarily because of their inability to generate interpretable parameters for each input variable. To mitigate this problem, sensitivity analysis was adapted to explain their inference mechanism[26]. In our study, each input variable to the network varied between the mean ± standard deviation, while all others were fixed at their respective means, and the corresponding change was recorded as a percentage deviation in the output. It could help illustrate the effect of changing a single input variable on the network output.

### ROC curves

The receive operating characteristic (ROC) methodology is a computational methodology which has a very important connection to the neural network applied to classification applications[27]. An important feature of the ROC curves is that they readily incorporate prevalence and misclassification cost factors in decision-making. In our study, the predictive accuracies of the neural network were tested by measuring the area under the ROC (AUROC). The optimal cutoff value to predict the absence or presence of significant fibrosis was chosen. Diagnostic accuracy was evaluated by calculating sensitivity, specificity, positive and negative predictive values (PPV and NPV), and Youden's index.

## Results

### Patients characteristics

From January 2006 to March 2008, 396 patients who showed evidence of HBsAg-positive and liver biopsy were investigated. Among them, 57 were excluded from this study because of the following reasons: 24 had HCV or HIV co-infection, 4 had acute hepatitis, 7 had hepatocellular carcinoma, and 22 lacked complete data on laboratory tests. The demographic and clinical characteristics were similar between those 57 excluded patients and the rest of 339 patients fulfilling the entry criteria. From April 2008, 116 CHB patients were considered for enrollment. So, a total of 455 patients were included in this study in the end. Of overall subjects, 174 (38.2%) had no fibrosis (F0), 155 (34.1%) had portal fibrosis (F1), 70 (15.4%) had septal fibrosis (F2), 47 (10.3%) had numerous septa (F3), and 9 (2.0%) had cirrhosis (F4). Their mean age was 33.9 ± 11.7 years; 289 (63.5%) were male, and 166 (36.5%) were female. Table 1 shows the comparison between patients with and without significant fibrosis. All markers, except for gender, hematoglobin (Hb), alkaline phosphatase (ALP) and total protein (TP), were found to be statistically significant factors associated with significant fibrosis, and were used to initially construct the artificial neural network.

**Table 1:** Comparing characteristics of patients with and without significant fibrosis

Variables	Patients without significant fibrosis (F0-1), n = 329	Patients with significant fibrosis (F2-4), n = 126	χ^2 ^value	P
Age	29(16-60)	40(17-75)	52.21	< 0.0001
Gender(M/F)	215/114	74/52	1.723	0.189
WBC(10^9^/L)	5.3(2.3-12.2)	4.5(1.3-18.1)	14.51	< 0.0001
RBC(10^12^/L)	4.8(3.5-4.0)	4.5(2.6-5.7)	9.47	0.002
Hb(g/L)	145(87-173)	141(85-167)	1.79	0.174
Platelet(10^9^/L)	184(46-350)	136(13-246)	80.24	< 0.0001
ALT(U/L)	72.0(6-471)	174.1(11-536)	11.37	< 0.001
AST(U/L)	15.2(11-341)	63.4(11-413)	28.43	< 0.0001
GGT(u/L)	24.9(6-473)	66.5(8-327)	62.14	< 0.0001
ALP(u/L)	106(7-219)	117(19-307)	2.78	0.099
ChE(u/L)	8054(70-20427)	6617(50-12648)	41.28	< 0.0001
TP(g/L)	72.3(49-88.4)	72.1(40-88.3)	0.442	0.501
Alb(g/L)	45.4(11.6-47.0)	43.2(10.9-52.6)	18.14	< 0.0001
TBIL(μmol/L)	13.4(3-138)	15.3(5.5-216)	11.03	< 0.0001
Length of liver biopsy(mm)	14(10-21)	12(11-19)	1.290	0.256
Number of liver biopsy	2.0(1.0-4.0)	2.5(1.0-5.0)	1.621	0.197

There were 226 subjects in the training set; 113 subjects in the validation set; 116 ubjects in the testing set. The variables used to construct the artificial neural network were compared in three sets (Table 2). These markers didn't appear to be statistically significant.

**Table 2:** Comparing input and output variables in three sets

Variables	Patients in the training set, n = 226	Patients in the validation set, n = 113	Patients in the testing set, n = 116	χ^2^(*p*)
Age(years)	30(16-62)	35(16-75)	32(16-74)	1.741(0.419)
WBC(10^9^/L)	5.4(1.3-18.1)	5.5(2.5-16.8)	5.4(3.0-11)	0.231(0.891)
RBC(10^12^/L)	4.7(2.6-4.0)	4.7(3.6-5.7)	4.6(3.7-5.6)	0.310(0.856)
Platelet(10^9^/L)	165(53-246)	170(13-350)	185(46-345)	4.965(0.083)
ALT(U/L)	87(16-471)	80(11-536)	72(6-507)	2.475(0.290)
AST(U/L)	48(11-413)	42.5(15-375)	39(11-361)	1.264(0.531)
GGT(U/L)	29(6-421)	33(6-288)	34(10-473)	3.247(0.197)
ChE(U/L)	8402(50-20427)	7674(145-11374)	7258(360-12648)	3.051(0.217)
Alb(g/L)	44.8(10.9-47.0)	44.2(11.8-50.7)	44.1(31.3-52.6)	0.412(0.814)
TBIL(μmol/L)	14.1(3.6-216)	15.3(3-196)	13.2(3- 134)	3.650(0.161)
Fibrosis F(0-1)/F(2-4)	166/60	83/30	80/36	0.657(0.720)

### Sensitivity analysis

**Figure 1:**
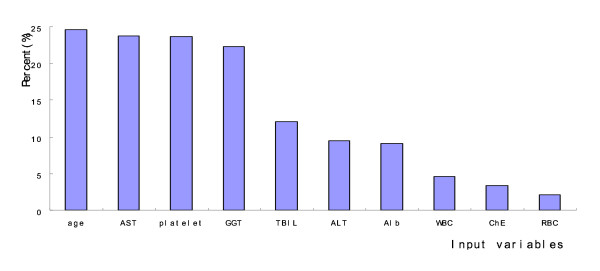
**Sensitivity analysis of input variables**. The horizontal axis is the input variables; the vertical axis is the percent change on the output variable. The value shown for each input variable is a measure of its relative importance, with 0 representing a variable that has no effect on the prediction and 1 representing a variable that completely dominates the prediction.

The sensitivity analysis of 10 variables was outlined in Figure 1. The value shown for each input variable is a measure of its relative importance, with 0 representing a variable that has no effect on the prediction and 1 representing a variable that completely dominates the prediction. The horizontal axis is the input variables; the vertical axis is the percent change on the output variable. The most important factors in the predictive model were age, aspartate aminotransferase (AST), platelet, and γ-glutamyltransferase (GGT), and the influence on the output variance among CHB patients were 24.6%, 23.8%, 23.7% and 22.3%, respectively.

### Accuracy of the ROC curves

**Figure 2:**
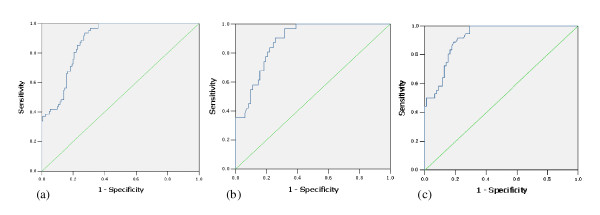
**ROC curve of the neural network output in three sets**. (a) In the training set(Cutoff = 0.415, AUROC = 0.883); (b) In the validation set(Cutoff = 0.421, AUROC = 0.884); (c) In the testing set(Cutoff = 0.418, AUROC = 0.920)

The performance of the neural network in predicting significant fibrosis in three sets was high, and the AUROC was as follows: 0.883 (in the training set), 0.884 (in the validation set), and 0.920 (in the testing set; Figure 2).

Sensitivity, specificity, positive and negative predictive values at different cutoff points in the testing set were shown in Table 3. For a decision threshold of 0.33, sensitivity and negative predictive values were 100%, which indicated that this model could predict insignificant fibrosis with the highest accuracy. This procedure would have correctly identified 47.4% of CHB patients without significant fibrosis, who needn't proceed with liver biopsy.

**Table 3:** ROC analysis of artificial neural network output at different cutoff points in the testing set

Cutoff point	Sensitivity%	Specificity%	PPV%	NPV%	Youden Index%
0.33	100.0	68.7	59.0	100.0	68.7
0.35	94.4	75.0	63.0	96.8	69.4
0.40	91.7	80.0	65.3	94.0	71.7
0.45	83.3	85.0	71.4	91.9	68.3
0.50	66.7	88.7	72.7	85.5	47.1
0.55	55.5	92.5	76.9	82.2	44.2
0.60	44.4	96.2	83.3	78.6	42.3
0.70	36.1	100.0	100.0	77.7	36.1

## Discussion

Liver fibrosis is characterized by the accumulation of an extracellular matrix (ECM), which distorts the hepatic architecture. Liver fibrosis progression could commonly be found in chronic hepatitis B patients after an extensive period of time during which liver biochemical indices are found to be predominately or even persistently abnormal[28]. Due to the limitation of liver biopsy, noninvasive evaluation of liver fibrosis is thus of great clinical interests in order to assess the risk for liver fibrosis dynamically, or identify and monitor the patients who should be considered antiviral or other types of therapy.

Age, platelet, AST, ALT, GGT, etc., as routine biochemical markers, had been well known predictors of significant liver fibrosis[14,29,30]. In the present study, based on common biochemical parameters including routine and serum markers, we constructed a three-layer neural network which extended a back-propagation learning algorithm by introducing probabilistic treatment of the Bayesian inference technique for the synaptic weight[31]. Results of sensitivity analysis showed the importance of various predictors. As could be seen, the most important variables influencing the prediction of significant liver fibrosis were age, AST, platelet and GGT. These results were consistent with some of the earlier studies[14,16]. Sensitivity analysis showed that the neural network using Bayesian approaches could achieve its predictive purpose.

As demonstrated by ROC curves, the predictive accuracy of the artificial neural network was reasonably high in the training, validation, and testing sets. The AUROC were 0.883, 0.884 and 0.920, respectively. In view with some noninvasive parameters directly or indirectly related to fibrogenesis, the most prevalent are the Fibrotest and the Actitest for necro-inflammator activity[32]. They are based on GGT, TBIL, haptoglobin, α2-macroglobulin, apolipoprotein A1, and for the Actitest additionally on ALT[33]. Previous studies on Fibrotest and Actitest have been validated with the ranges of the AUROC of 0.75- 0.89 in CHB patients[14,25,32,34]. Although the two indices could provide better predictive values according to different criteria, they were calculated with a patented and complicated algorithm, and it was difficult for physicians to use them to identify the states of liver fibrosis.

Hui AY constructed and validated a multivariate logistic regression model using body mass index, platelet, Alb, and TBIL level to predict advanced fibrosis, and the AUROC were 0.765-0.803[17]. Zeng MD also constructed a scoring system with forward logistic regression, which was expressed by the following formula[16]: -13.995 + 3.22l g(α_2_-macroglobulin)+ 3.096 lg(age) + 2.254 lg(GGT) + 2.437 lg(HA). The AUROC of this model in the training and validation groups were 0.84 and 0.77, respectively. The logistic regression, a generalized linear model used for binomial regression, is used for prediction of the probability of occurrence of an event by fitting data to a logistic curve. The artificial neural networks, a non-linear statistical data modeling tool, can be used to model complex relationships between inputs and outputs. Therefore, the present study provided evidence that the three-layer neural network model based on routine and serum markers was superior to other indices or models for identification of individual with or without significant fibrosis.

In our study, there were 126 patients with significant liver fibrosis, which only accounted for 27.7% of 455 CHB patients with liver biopsy. In other words, 329 (72.3%) patients without significant liver fibrosis had undergone liver biopsy, and they could bear the damages from such an invasive procedure. From a more practical point of view, we wanted to reduce the number of liver biopsy procedures and also identify all CHB patients with significant fibrosis. Therefore, our study evaluated the influence of the different cutoff points on the accuracy of ROC. When we chose a high cutoff point, the number of CHB patients at high risk for significant liver fibrosis was few, and also fewer patients needed further liver biopsy or other examinations. But, there was a low sensitivity and many CHB patients with significant liver fibrosis could be missed. The purpose of predicting the state of liver fibrosis is to identify CHB patients with significant liver fibrosis or at high risk for liver fibrogenesis to prevent them from further liver fibrogenesis. Thus, we should choose a lower cutoff point to improve the sensitivity, and to reduce the number of missed CHB patients at high risk for liver fibrogenesis. So, we considered a probability value of 0.33 as a cutoff value. CHB patients with a probability value > 0.33 were considered with significant liver fibrosis or at high risk for liver fibrogenesis. In our study, all CHB patients with significant liver fibrosis would be identified. 47.4% (55/116) of the CHB patients would be free of liver biopsy and also wouldn't be missed.

## Limitations of the study

Our study was conducted in a specialized hospital for infectious diseases. All individuals investigated were in-patients, therefore not a completely random sample of all CHB patients. However, owing to the limitation of liver biopsy, we thought that the predictive model in this study might be applied to identify the risk of liver fibrosis as some studies showed [35].

The accuracy of a test could vary with the definition of the target condition[36]. In our study, the occurrence of F2, F3, or F4 was considered as significant fibrosis so that the prevalence of significant fibrosis was relatively low, which could affect the diagnostic accuracy of the model by AUROC. The DANA (difference of prevalence of advanced and nonadvanced fibrosis stages) was used for the standardization of AUROC of fibrosis marker or model[37]. But comparing with other studies [14,16,17,25,32], we didn't correct the AUROC by the DANA.

## Conclusions

The study highlighted the construction and assessment of an artificial neural network for identifying the risk for liver fibrosis. Age, platelet, AST, ALT, GGT, etc., as the input variables of the artificial neural network model, were widely available. The results of our study showed that the three-layer artificial neural networks could effectively identify the risk for liver fibrosis in CHB patients with positive HBsAg. It could improve patient compliance, and reduce the need for liver biopsy required prior to antiviral or other types of therapy.

## Abbreviations

HBV: hepatitis B virus; CHB: chronic hepatitis B; HCC: hepatocellular; ANN: artificial neural network; HBsAg-positive: hepatitis B surface antigen; CHC: chronic hepatitis C; HE -hepatitis E; HCV: hepatitis C virus; HIV: human immunodeficiency virus; ROC: receive operating characteristic; AUROC: area under ROC; PPV: positive predictive values; NPV: negative predictive values; ECM: carcinoma extracellular matrix; WBC: white blood cell; RBC: red blood cell; Hb: hematoglobin; ALT: alanine aminotransferase; AST: aspartate aminotransferase; GGT: γ-glutamyltransferase; ALP: alkaline phosphatase; ChE: Cholinesterase; TP: total protein; Alb: albumin; TBIL: total bilirubin

## Competing interests

The authors declare that they have no competing interests.

## Authors' contributions

DW conducted the study, participated in the data collection and wrote the initial draft and revised the manuscripts. QW collected the preliminary data, and helped to draft the manuscript. FS participated in the study design and interpretation of the data. BL participated in data collection and gave inputs to the drafting of the manuscript. CL helped to coordinate the study and the drafting and revisions of the paper. All co-authors read and approved the final manuscript.

## Pre-publication history

The pre-publication history for this paper can be accessed here:

http://www.biomedcentral.com/1471-2334/10/251/prepub
